# Up-regulation of telomerase activity in human pancreatic cancer cells after exposure to etoposide

**DOI:** 10.1054/bjoc.2000.1117

**Published:** 2000-06

**Authors:** N Sato, K Mizumoto, M Kusumoto, S Nishio, N Maehara, T Urashima, T Ogawa, M Tanaka

**Affiliations:** Department of Surgery and Oncology, Graduate School of Medical Sciences, Kyushu University, Fukuoka, 812-8582, Japan

**Keywords:** telomerase, topoisomerase II inhibitor, DNA damage, hTERT, apoptosis, drug resistance

## Abstract

Telomerase plays a critical role in the development of cellular immortality and oncogenesis. Activation of telomerase occurs in a majority of human malignant tumours, and the relation between telomerase and vulnerability to drug-mediated apoptosis remains unclear. In this study, we demonstrate, for the first time, up-regulation of telomerase activity in human pancreatic cancer cells treated with etoposide, a topoisomerase II inhibitor. Exposure of MIA PaCa-2 cells to etoposide at various concentrations (1–30 μM) resulted in two- to threefold increases in telomerase activity. Up-regulation was detectable 24 h after drug exposure and was accompanied by enhanced expression of mRNA of the human telomerase reverse transcriptase. Telomerase activation was also observed in AsPC-1 and PANC-1 cells but not in KP-3 and KP-1N cells. Furthermore, we found a negative correlation between increased telomerase activity and the percentage of dead cells after etoposide treatment. These findings suggest the existence of an anti-apoptotic pathway through which telomerase is up-regulated in response to DNA damage. This telomerase activation pathway may be one of the mechanisms responsible for the development of etoposide resistance in certain pancreatic cancer cells. © 2000 Cancer Research Campaign


*British Journal of Cancer* (2000) **82**(11), 1819–1826
© 2000 Cancer Research Campaign
DOI: 10.1054/ bjoc.2000.1117, available online at http://www.idealibrary.com on IDEAL

### Up-regulation of telomerase activity in human pancreatic cancer cells after exposure to etoposide

**N Sato, K Mizumoto, M Kusumoto, S Nishio, N Maehara, T Urashima, T Ogawa and M Tanaka**

Department of Surgery and Oncology, Graduate School of Medical Sciences, Kyushu University, Fukuoka 812-8582, Japan

**Summary** Telomerase plays a critical role in the development of cellular immortality and oncogenesis. Activation of telomerase occurs in a majority of human malignant tumours, and the relation between telomerase and vulnerability to drug-mediated apoptosis remains unclear. In this study, we demonstrate, for the first time, up-regulation of telomerase activity in human pancreatic cancer cells treated with etoposide, a topoisomerase II inhibitor. Exposure of MIA PaCa-2 cells to etoposide at various concentrations (1–30 μM) resulted in two- to threefold increases in telomerase activity. Up-regulation was detectable 24 h after drug exposure and was accompanied by enhanced expression of mRNA of the human telomerase reverse transcriptase. Telomerase activation was also observed in AsPC-1 and PANC-1 cells but not in KP-3 and KP-1N cells. Furthermore, we found a negative correlation between increased telomerase activity and the percentage of dead cells after etoposide treatment. These findings suggest the existence of an anti-apoptotic pathway through which telomerase is up-regulated in response to DNA damage. This telomerase activation pathway may be one of the mechanisms responsible for the development of etoposide resistance in certain pancreatic cancer cells. © 2000 Cancer Research Campaign

**Keywords**: telomerase; topoisomerase II inhibitor; DNA damage; hTERT; apoptosis; drug resistance

Despite tremendous efforts in early diagnosis and therapy, the prognosis for patients with pancreatic carcinoma remains dismal (Warshaw and Fernandez-del Castillo, 1992; Niederhuber et al, 1995). In advanced pancreatic cancer, drug resistance is a major obstacle to chemotherapy (Arbuck, 1990; Lionetto et al, 1995; Schnall and Macdonald, 1996). Considerable evidence shows that apoptosis plays a key role in tumour cell death induced by several chemotherapeutic agents (Kerr et al, 1994; Huschtscha et al, 1996). Tumour cells may express defects in the apoptotic pathway and become resistant to chemotherapy (Thompson, 1995; Hickman, 1996; Reed et al, 1996). A better understanding of the mechanisms underlying the resistance to drug-mediated apoptosis may enhance our ability to treat pancreatic cancer successfully.

Telomerase, a ribonucleoprotein enzyme, elongates and maintains telomeric DNA through the addition of TTAGGG repeats and plays a critical role in the development of cellular immortality and carcinogenesis (Morin, 1989; Harley et al, 1994; Kim et al, 1994; Shay and Bacchetti, 1997). We previously reported highly elevated telomerase activity in 80% of specimens from surgically resected lesions of pancreatic carcinoma and in 75% of pancreatic juice samples obtained from patients with ductal carcinoma (Suehara et al, 1997*a*, 1997*b*). Three major components of human telomerase have been identified: human telomerase RNA, hTERC (Feng et al, 1995); telomerase-associated protein, TEP1 (Harrington et al, 1997; Nakayama et al, 1997); and human telomerase reverse transcriptase, hTERT (Meyerson et al, 1997; Nakamura et al, 1997). Among these components, hTERT has been considered a catalytic subunit of telomerase and a rate-limiting determinant of the enzymatic activity (Bodnar et al, 1998; Vaziri and Benchimol, 1998).

Although telomerase may be implicated in the cellular response to apoptosis, the relation between telomerase and vulnerability to apoptosis remains poorly understood. Antisense inhibition of telomerase increases the susceptibility of glioblastoma cells to cisplatin-induced apoptosis (Kondo et al, 1998). Similarly, telomerase inhibition with the oligodeoxynucleotide enhances apoptosis in pheochromocytoma cells induced by a variety of stimuli (Fu et al, 1999). Enhanced expression of telomerase and maintenance of telomere stability increase cellular resistance to apoptosis triggered by serum starvation or matrix deprivation (Holt et al, 1999). These findings suggest a protective role of telomerase against apoptotic cell death. We hypothesized that telomerase activity could be one determinant of vulnerability to drug-induced apoptosis. Studies on telomerase regulation in cancer cells treated with antineoplastic agents could elucidate the functional role of this enzyme in drug-induced apoptosis.

In the present study, we investigated the dynamics of telomerase activity in human pancreatic cancer cells treated with etoposide, which is known to cause DNA strand breaks and to induce apoptosis in diverse cell types (Barry et al, 1993; Mizumoto et al, 1994; Okamoto-Kubo et al, 1994; Bonelli et al, 1996; Lock and Stribinskiene, 1996; Hande, 1998). We found increased telomerase activity after etoposide exposure in cell lines that showed resistance to etoposide-induced apoptosis. To the best of our knowledge, this is the first report describing up-regulation of telomerase activity in response to etoposide. Our results support the hypothesis that telomerase plays a protective role against DNA damage and subsequent apoptosis through its up-regulation pathway.

*Received 15 November 1999*
*Revised 1 February 2000*
*Accepted 3 February 2000*

*Correspondence to:* K Mizumoto

### MATERIALS AND METHODS

### Cell lines and culture conditions

Five human pancreatic cancer cell lines were used in this study: MIA PaCa-2 was provided by the Japanese Cancer Resource Bank (Tokyo, Japan); AsPC-1, PANC-1, KP-3, and KP-1N were donated by Dr Iguchi (National Kyushu Cancer Center, Fukuoka, Japan). Cells were maintained in Dulbecco's modified Eagle's medium (Sigma Chemical Co., St Louis, MO, USA) supplemented with 10% fetal bovine serum, streptomycin (100 μg $ml^{-1}$), and penicillin (100 U $ml^{-1}$) at 37°C in a humidified atmosphere containing 10% carbon dioxide.

### Drug treatment

Etoposide (Nippon Kayaku Co., Ltd., Tokyo, Japan) was dissolved in dimethylsulphoxide (DMSO) at 10 mM as a stock solution. Cells in log phase growth were seeded at a density of $5 \times 10^4$ cells $well^{-1}$ in 12- or 24-well plates. After overnight incubation, the cells were exposed continuously to etoposide at various concentrations. Cell number and mean cell diameter were measured with a particle distribution analyser, CDA500 (Sysmex, Kobe, Japan). Five days after the beginning of treatment, cell viability was assessed with the cell-death assay described below.

### Measurement of DNA synthesis

DNA synthesis was determined with the [$^3$H]thymidine incorporation test. Cells were exposed to etoposide (5 μM) for 24 h and pulsed for 4 h with 1 μCi $ml^{-1}$ of [$^3$H]thymidine. The incorporation of [$^3$H]thymidine into trichloroacetic acid-insoluble material was measured with a liquid scintillation counter.

### Cell cycle analysis

Cells were harvested at indicated time points, washed twice in phosphate-buffered saline (PBS) and fixed with cold 70% ethanol for 4 h at 4°C. Fixed cells were then washed with PBS, incubated with RNAase (100 μg $ml^{-1}$) for 30 min at room temperature, stained with propidium iodide (PI, Sigma, 50 μg $ml^{-1}$) and analysed on a FACScan flow cytometer (Becton Dickinson, Bedford, MA, USA).

### Assessment of apoptosis

After drug treatment, detached cells were combined with monolayer cells that had been detached by trypsinization and applied to glass slides with cytospin preparations. Cells were fixed in cold methanol, and membranes were permeabilized with 0.2% Triton X-100 (Sigma) and stained with DNA dye, PI. Nuclear morphology was observed under a laser-scanning microscope, Olympus LSM-GB200 system (Tokyo, Japan).

### Quantitative measurement of dead cells

Dead cells were measured quantitatively with a multiwell fluorescence scanner by the method described previously (Sato et al, 1998). This method is based on the binding of PI to the nuclei of cells with plasma membranes that are permeable as a result of cell death. The percentage of dead cells was defined as the proportion of fluorescence intensity corresponding to dead cells to that of total cells.

### Telomerase activity assay

Telomerase activity was measured based on a telomerase repeat amplification protocol (TRAP) assay (Kim et al, 1994) performed with a TRAPeze Telomerase Detection Kit (Intergen, Purchase, NY, USA) according to the manufacturer's instructions. For a sensitive and semiquantitative telomerase assay, cell extracts were diluted to a concentration equivalent to a constant number of cells for each assay. PCR products were resolved by electrophoresis on a 12% polyacrylamide gel and visualized with SYBR Green DNA stain (FMC Bioproducts, Rockland, ME, USA). A 36-bp internal standard was used as an internal control. For quantification of telomerase activity in each sample, the total density of the entire ladder was measured with NIH-Image software (NTIS, Springfield, VA, USA).

### RT-PCR analysis

Analysis of the mRNA expression of hTERT, TEP1 and hTERC was performed by reverse transcription polymerase chain reaction (RT-PCR) amplification. An Isogen RNA extraction kit (Nippon Gene, Tokyo, Japan) was used to isolate total RNA from the cell pellets. cDNA was synthesized from 2 μg of RNA with the RNA PCR kit (Takara Shuzo Co. Ltd, Otsu, Japan) and random primers. Two-microlitre aliquots of the reverse-transcribed cDNA were subjected to PCR amplification with an initial incubation step at 94°C for 3 min, followed by 33 cycles at 94°C for 45 s, 60°C for 45 s and 72°C for 90 s. The primer pairs were as follows: 5′-TGAGTGTGTACGTCGTCGAG-3′ (sense) and 5′-GAACG-TTCTGGCTCCCACGA-3′ (antisense) for hTERT; 5′-TCA-AGCCAAACCTGAATCTGAG-3′ (sense) and 5′-CCCCGAGT-GAATCTTTCTACGC-3′ (antisense) for TEP1; and 5′-TCTAAC-CCTAACTGAGAAGGGCGTAG-3′ (sense) and 5′-GTTTGCT-CTAGAATCAACGGTGGAAG-3′ (antisense) for hTERC. The PCR products were separated on a 2% agarose gel and visualized by ethidium bromide staining.

### Statistical analysis

The results of parametric data are expressed as means ± standard deviation (s.d.). Correlation between the magnitude of telomerase up-regulation and the percentage of dead cells was assessed with the Spearman's rank correlation test. $P < 0.05$ was considered significant.

### RESULTS

### Cytotoxic effect of etoposide on MIA PaCa-2 cells

We first evaluated the cytotoxicity of etoposide on MIA PaCa-2 cells. Control cells were treated with solvent (DMSO) alone. Treatment with etoposide (5 μM) completely inhibited the growth of MIA PaCa-2 cells (Figure 1A). DNA synthesis, determined by the [$^3$H]thymidine incorporation test, decreased after etoposide exposure. The [$^3$H]thymidine uptake in cells treated with 5 μM etoposide for 24 h was 41.8 ± 0.3% that of control cells. Flow cytometric analysis revealed that the cells were arrested at the G2/M phase 48 h after etoposide treatment. In control cells, the

 © 2000 Cancer Research Campaign

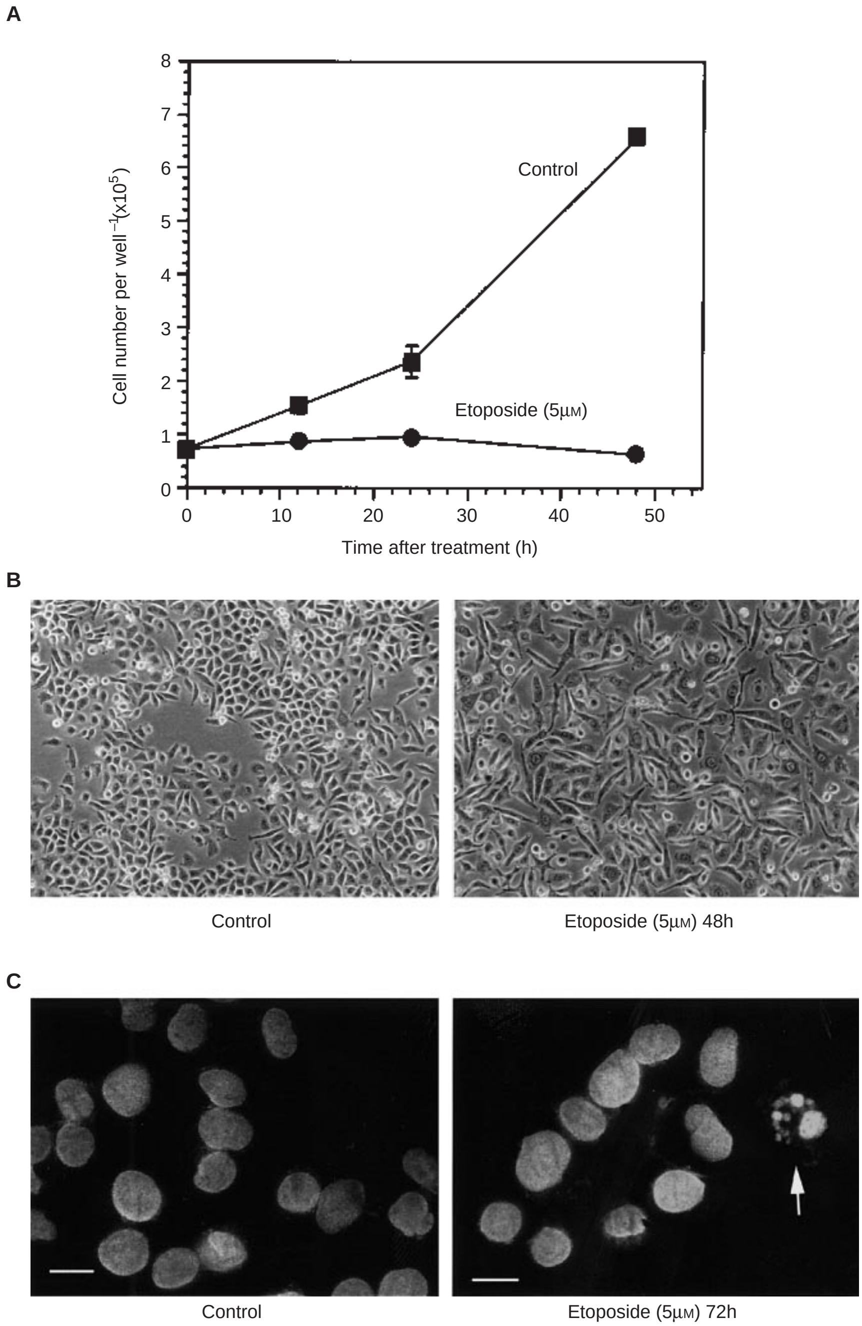

**Figure 1** Cytotoxic effect of etoposide on MIA PaCa-2 cells. (**A**) Growth kinetics of MIA PaCa-2 cells treated with solvent alone (closed square) or 5 µM etoposide (closed circle). Cell numbers are means ± s.d. of three independent wells. (**B**) Phase contrast photomicrographs of MIA PaCa-2 cells exposed to solvent (left panel) or 5 µM etoposide (right panel) for 48 h; ×100. (**C**) Nuclear morphology of MIA PaCa-2 cells 72 h after treatment with solvent (left panel) or 5 µM etoposide (right panel) stained with propidium iodide. Apoptotic cells manifesting nuclear fragmentation are observed in a small fraction of etoposide-treated cells (arrow). Bars, 20 µm phase values were 50% for G0/G1, 39% for S, and 11% for G2/M. In cells treated with etoposide for 48 h, the values were 21% for G0/G1, 22% for S, and 57% for G2/M. Morphologically, cells exposed to etoposide were enlarged with a mean cell diameter of 24.7 ± 3.7 µm 24 h after treatment compared to 21.3 ± 3.0 µm in control cells (Figure 1B). Apoptotic cell death, characterized by cell shrinkage, plasma membrane blebbing, chromatin condensation and nuclear fragmentation, was detectable in a small number of cells beginning on day 3 (Figure 1C). The percentage of dead cells in the culture treated with 5 µM etoposide was 7.7 ± 1.2% on day 5. Almost no cell death was observed in solvent-treated cells (data not shown).

### Up-regulation of telomerase activity in MIA PaCa-2 cells treated with etoposide

To determine an appropriate concentration (cell number equivalent) of cell extract that would permit reliable quantification of

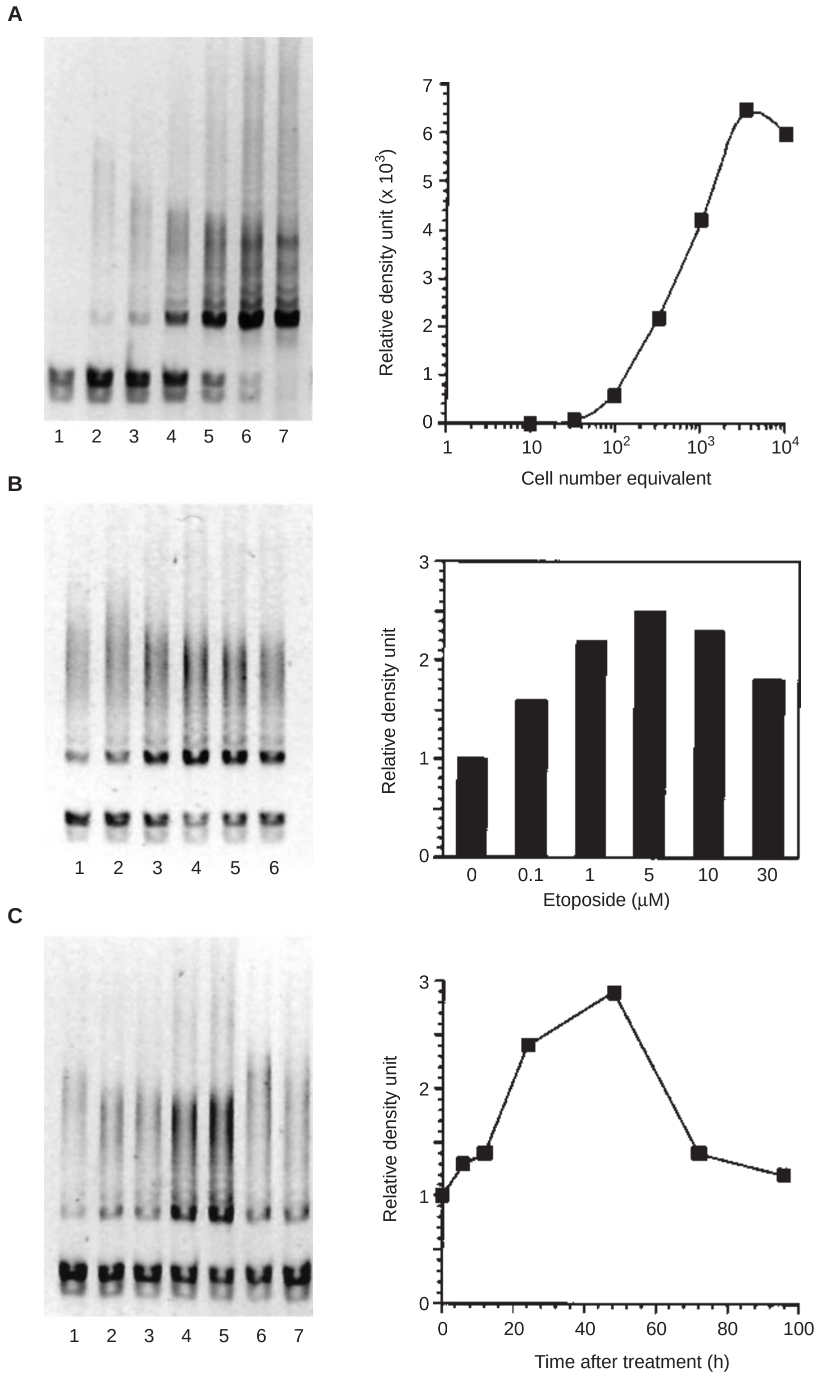

**Figure 2** Up-regulation of telomerase activity in MIA PaCa-2 cells treated with etoposide. (**A**) Telomerase activity in serial dilution of cellular extracts, corresponding to 10 (lane 1), 30 (lane 2), 100 (lane 3), 300 (lane 4), 1000 (lane 5), 3000 (lane 6) and 10 000 (lane 7) cells. Data points, relative density unit of telomerase ladders in each lane. Linearity of the ladder density was observed between 100 and 3000 cell equivalents. (**B**) Telomerase activity in cells treated for 24 h with etoposide at a concentration of 0 (control, lane 1), 0.1 (lane 2), 1 (lane 3), 5 (lane 4), 10 (lane 5) and 30 μM (lane 6). Columns, relative density of telomerase ladders to that of control cells. (**C**) Telomerase activity in cells treated with etoposide for 0 (control, lane 1), 6 (lane 2), 12 (lane 3), 24 (lane 4), 48 (lane 5), 72 (lane 6) and 96 h (lane 7) at a concentration of 5 μM. Data points, relative density of telomerase ladders to that of control cells relative levels of telomerase activity, the basal level of telomerase activity was measured in a serial dilution of the MIA PaCa-2 cell extract. One hundred to 3000 cell equivalents were within linear range of the relative telomerase activity (Figure 2A). In subsequent assays, we applied extracts equivalent to $10^2$ cells to precisely detect differences in telomerase activity. Twenty-four hours after treatment with etoposide at various (0.1–30 μM) concentrations, we examined MIA PaCa-2 cells for changes in telomerase activity. Etoposide significantly enhanced telomerase activity of treated cells even at relatively low doses (Figure 2B). The maximum telomerase up-regulation was obtained at a concentration of 5 μM. A time course experiment of telomerase activity in

 *© 2000 Cancer Research Campaign*

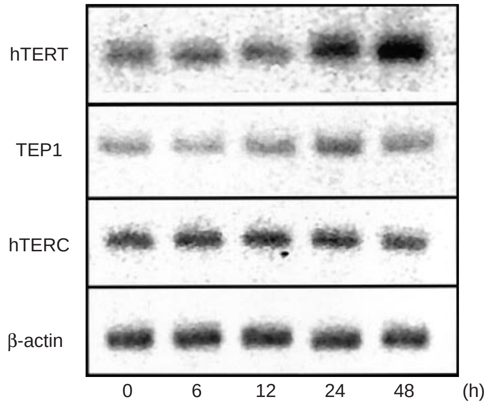

**Figure 3** RT-PCR analysis of mRNA expression of hTERT, TEP1, hTERC and β-actin (as a control) in MIA PaCa-2 cells treated with etoposide for 0, 6, 12, 24 and 48 h at a concentration of 5 μM. A marked increase is apparent in the mRNA expression of hTERT, whereas no significant change is detectable in those of TEP1 and hTERC

MIA PaCa-2 cells treated with 5 μM etoposide revealed elevated levels as early as 24 h after treatment began and increases up to approximately threefold the control level at 48 h (Figure 2C). After 72 h, telomerase activity decreased gradually and returned to the pretreatment level at 96 h. When etoposide was added directly to MIA PaCa-2 cell extracts immediately before primer addition, telomerase activity remained unchanged (data not shown). Thus, a direct influence of etoposide on TRAP assay does not seem to be involved in the telomerase activation observed after drug treatment.

### Effect of etoposide on mRNA expression of hTERT, TEP1 and hTERC in MIA PaCa-2 cells

We also examined the effect of etoposide on the mRNA expressions of three telomerase subunits, hTERT, TEP1 and hTERC by RT-PCR. MIA PaCa-2 cells treated with etoposide (5 μM) displayed a substantial increase in hTERT mRNA expression (Figure 3). The increased level was detectable at 24 h, peaked at 48 h after drug exposure, and paralleled the up-regulation of telomerase activity encountered in the treated cells. In contrast, no significant increase was observed in TEP1 and hTERC mRNA levels during etoposide treatment. These results suggest that hTERT plays a critical role in the regulation of telomerase activity and that etoposide up-regulates telomerase activity of treated cells through induction of hTERT mRNA expression.

**Table 1** The magnitude of telomerase up-regulation and cell death in pancreatic cancer cell lines treated with etoposide

| Cell line | Fold increase in telomerase activity[a] | Percentage dead cells (mean ± s.d.)[b] |
|---|---|---|
| MIA PaCa-2 | 2.9 | 7.7 ± 1.2% |
| AsPC-1 | 3.1 | 2.5 ± 0.8% |
| PANC-1 | 2.2 | 21.8 ± 2.7% |
| KP-3 | 1.3 | 37.5 ± 5.4% |
| KP-1N | 1.1 | 54.5 ± 0.8% |

[a]Ratio of the highest telomerase activity in cells treated with etoposide (5 μM) to that of control cells. [b]Percentage of dead cells 5 days after treatment with etoposide (5 μM).

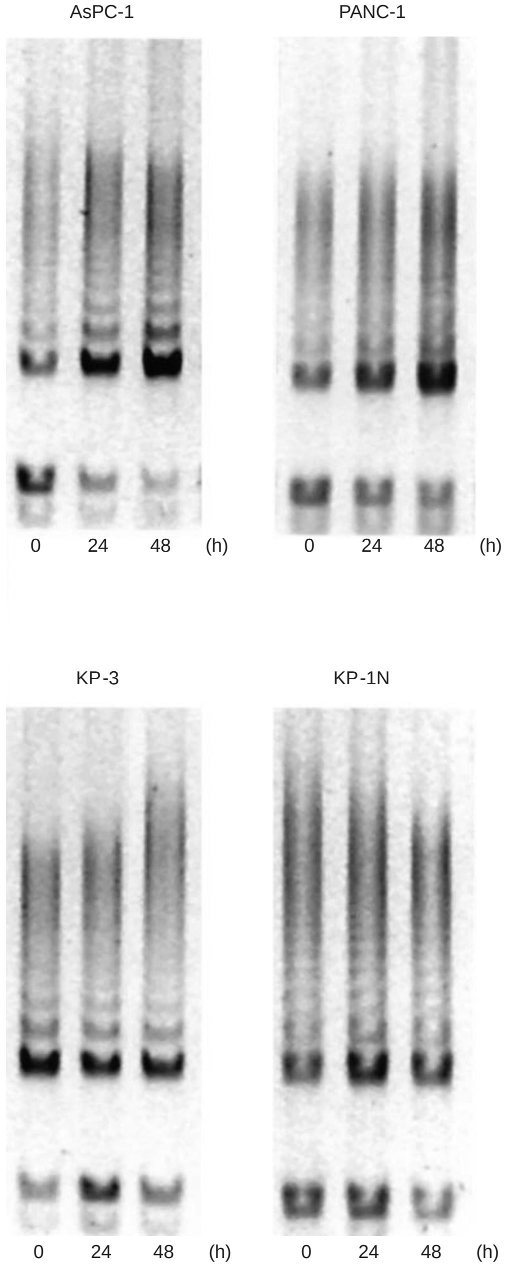

**Figure 4** Effect of etoposide on telomerase activity in four pancreatic cancer cell lines. Cells were treated with etoposide (5 μM) for 24 h and 48 h, and telomerase activity was assessed. Telomerase up-regulation is shown in AsPC-1 and PANC-1 cells but not in KP-3 and KP-1N cells

### Relation between telomerase up-regulation and sensitivity to etoposide-induced cell death

Other cell lines were treated with etoposide at a concentration of 5 μM and assayed for telomerase activity in the same manner. AsPC-1 and PANC-1 cells exhibited an increase in telomerase activity, whereas KP-3 and KP-1N cells showed no significant change after etoposide treatment (Figure 4 and Table 1). Since telomerase is implicated in the anti-apoptotic phenotypes, we attempted to determine whether a correlation exists between telomerase activity and etoposide-induced apoptosis. MIA PaCa-2, AsPC-1 and PANC-1, all of which showed telomerase elevation,

© 2000 Cancer Research Campaign

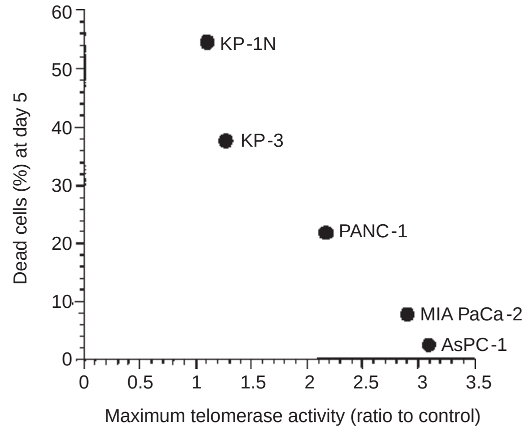

**Figure 5** Relation between the magnitude of telomerase up-regulation and vulnerability to etoposide-induced apoptosis in five pancreatic cancer cell lines. There is a significant negative correlation between maximum telomerase activity (ratio to control) upon exposure to etoposide and percentage of dead cells at day 5 ($P < 0.05$)

were relatively resistant to etoposide-mediated apoptosis. The percentage of dead cells at day 5 was 7.7 ± 1.2% in MIA PaCa-2, 2.5 ± 0.8% in AsPC-1, and 21.8 ± 2.7% in PANC-1 (Table 1). KP-3 and KP-1N, with unchanged telomerase activity, were sensitive to etoposide-induced lethality. The proportion of dead cells was 37.5 ± 5.4% in KP-3 and 54.5 ± 0.8% in KP-1N. The magnitudes of telomerase up-regulation (the ratio of the highest telomerase activity to that of control cells) in these five lines were significantly correlated with the percentages of dead cells ($P < 0.05$; Figure 5). These findings indicate that some types of pancreatic cancer cells may escape etoposide-induced apoptotic crisis via the mechanism of telomerase activation.

### DISCUSSION

The aim of this study was to investigate the effect of etoposide on telomerase activity and to clarify the relation between telomerase dynamics and etoposide-mediated apoptotic cell death. The results demonstrate that etoposide enhances telomerase activity in human pancreatic cancer cells by including its catalytic subunit hTERT mRNA expression. Our findings have implications for understanding the potential role of telomerase in cancer cells.

Our present data conflict with previous studies that found no significant change in telomerase activity in leukaemic cells and nasopharyngeal carcinoma cells after etoposide treatment (Ku et al, 1997; Akiyama et al, 1999). Direct comparison of these studies and ours is difficult because of differences in cell lines used and in methodologies. Telomerase plays an essential role in the maintenance of genomic integrity (Lee et al, 1998), and its activation upon exposure to etoposide could result from the need of cells to repair DNA. This idea is supported by a study describing the stabilization of broken chromosomes by the direct addition of telomeric repeats to nontelomeric DNA (Flint et al, 1994). Healing process of broken DNA might be mediated by telomerase. Consistent with our present results, a previous report showed up-regulation of telomerase activity in haematopoietic cells after exposure to ionizing radiation (Leteurtre et al, 1997).

Factors suggested to influence telomerase activity include cellular proliferation, cell cycle progression, differentiation, cellular senescence and apoptosis. Telomerase activity is associated with cell proliferation (Belair et al, 1997). However, our data show that etoposide inhibits the growth and DNA synthesis of treated cells rather than promotes their proliferation. The relation between telomerase activity and cell cycle is controversial (Holt et al, 1997). During cell cycle progression, telomerase activity has been noted as highly activated in the S phase and barely detectable in the G2/M phase (Zhu et al, 1996). We found that etoposide arrested most cells at the G2/M phase of the cell cycle by 48 h after treatment, when the telomerase up-regulation was maximal. Telomerase elevation in response to etoposide may not be related to the cell cycle-dependent modulation of this enzyme. Telomerase activity is also modulated by apoptosis. When tumour cell lines were treated with antineoplastic agents, including cisplatin and adriamycin, drug-induced cell killing was associated with a decline in telomerase activity (Faraoni et al, 1997). Apoptotic cells with an intact plasma membrane were reported to maintain high telomerase activity, whereas cells with plasma membrane damage lost telomerase activity (Akiyama et al, 1999). In our present study, telomerase elevation was detectable as early as 24 h after etoposide exposure, prior to the appearance of apoptotic cell death. After 72 h, telomerase activity slowly decreased, which is consistent with the loss of enzymatic activity resulting from apoptotic cell death. Etoposide-mediated telomerase activation may be a relatively early event that is independent of cell proliferation, cell cycle and apoptotic cell death.

We also examined the linkage between telomerase activity and vulnerability to etoposide-induced apoptosis. Although some investigators have suggested an anti-apoptotic role of telomerase in different cell types, the functional role of this enzyme in the drug-induced apoptosis remains poorly understood. We did not observe a significant relation between the basal level of telomerase activity in individual lines and their susceptibilities to etoposide-induced apoptosis (results not shown). We did find a negative correlation between increased telomerase activity and the percentage of dead cells after etoposide treatment. These findings raise the possibility that the elevated activity of telomerase can confer a survival advantage upon tumour cells and contribute to their decreased sensitivity to etoposide-mediated apoptosis. Detection of telomerase elevation, rather than the basal level of the enzyme, could be efficacious in determining the potential success of chemotherapy.

Susceptibility to drug-induced apoptosis is modulated by various genetic and biochemical alterations. For example, *p53* tumour suppressor gene product is essential for proper induction of apoptosis (Lowe et al, 1993, 1994). Tumour cells harbouring mutant p53 are generally more resistant to DNA damaging agents, such as etoposide, than cell expressing wild-type *p53* (Fan et al, 1994; Perego et al, 1996; O'Connor et al, 1997). Regarding the *p53* gene status, all the lines used in this study have *p53* mutations except for KP-3 (Iguchi et al, 1994). Thus, *p53* alone can not explain the different responses to etoposide-induced cell killing between these cell lines. Several complex factors, such as other genes controlling apoptosis, may be involved in the mechanisms underlying drug resistance of cancer cells. Our present results suggest that telomerase activation might be one of the key events responsible for the development of drug resistance in certain tumour cell types.

In conclusion, we observed increased telomerase activity after etoposide exposure. Although further studies will be required to delineate the precise mechanism by which telomerase activity is

 © 2000 Cancer Research Campaign up-regulated in response to DNA damage, our data suggest that a close linkage exists between vulnerability to drug-mediated apoptosis and activation of telomerase in human pancreatic cancer cells. A targeted blockage of this telomerase elevation pathway could be a potential anticancer strategy for sensitizing tumour cells to certain antineoplastic agents.

### REFERENCES

Akiyama M, Horiguchi-Yamada J, Saito S, Hoshi Y, Yamada O, Mizoguchi H and Yamada H (1999) Cytostatic concentrations of anticancer agents do not affect telomerase activity of leukaemic cells in vitro. *Eur J Cancer* **35**: 309–315

Arbuck SG (1990) Overview of chemotherapy for pancreatic cancer. *Int J Pancreatol* **7**: 209–222

Barry MA, Reynolds JE and Eastman A (1993) Etoposide-induced apoptosis in human HL-60 cells is associated with intracellular acidification. *Cancer Res* **53**: 2349–2357

Belair CD, Yeager TR, Lopez PM and Reznikoff CA (1997) Telomerase activity: a biomarker of cell proliferation, not malignant transformation. *Proc Natl Acad Sci USA* **94**: 13677–13682

Bodnar AG, Ouellette M, Frolkis M, Holt SE, Chiu CP, Morin GB, Harley CB, Shay JW, Lichtsteiner S and Wright WE (1998) Extension of life-span by introduction of telomerase into normal human cells. *Science* **279**: 349–352

Bonelli G, Sacchi MC, Barbiero G, Duranti F, Goglio G, Verdun di Cantogno L, Amenta JS, Piacentini M, Tacchetti C and Baccino FM (1996) Apoptosis of L929 cells by etoposide: a quantitative and kinetic approach. *Exp Cell Res* **228**: 292–305

Fan S, El-Deiry WS, Bae I, Freeman J, Jondle D, Bhatia K, Fornace AJ Jr, Magrath I, Kohn KW and O'Connor PM (1994) *p53* gene mutations are associated with decreased sensitivity of human lymphoma cells to DNA damaging agents. *Cancer Res* **54**: 5824–5830

Faraoni I, Turriziani M, Masci G, De Vecchis L, Shay JW, Bonmassar E and Graziani G (1997) Decline in telomerase activity as a measure of tumor cell killing by antineoplastic agent in vitro. *Clin Cancer Res* **3**: 579–585

Feng J, Funk WD, Wang SS, Weinrich SL, Avilion AA, Chiu CP, Adams RR, Chang E, Allsopp RC, Yu J, Le S, West MD, Harley CB, Andrews WH, Greider CW and Villeponteau B (1995) The RNA component of human telomerase. *Science* **269**: 1236–1241

Flint J, Craddock CF, Villegas A, Bentley DP, Williams HJ, Galanello R, Cao A, Wood WG, Ayyub H and Higgs DR (1994) Healing of broken human chromosomes by the addition of telomeric repeats. *Am J Hum Genet* **55**: 505–512

Fu W, Begley JG, Killen MW and Mattson MP (1999) Anti-apoptotic role of telomerase in pheochromocytoma cells. *J Biol Chem* **274**: 7264–7271

Hande KR (1998) Etoposide: four decades of development of a topoisomerase II inhibitor. *Eur J Cancer* **34**: 1514–1521

Harley CB, Kim NW, Prowse KR, Weinrich SL, Hirsch KS, West MD, Bacchetti S, Hirte HW, Counter CM, Greider CW, Piatyszek MA, Wright WE and Shay JW (1994) Telomerase, cell immortality, and cancer. *Cold Spring Harb Symp Quant Biol* **59**: 307–315

Harrington L, McPhail T, Mar V, Zhou W, Oulton R, Bass MB, Arruda I and Robinson MO (1997) A mammalian telomerase-associated protein. *Science* **275**: 973–977

Hickman JA (1996) Apoptosis and chemotherapy resistance. *Eur J Cancer* **32A**: 921–926

Holt SE, Aisner DL, Shay JW and Wright WE (1997) Lack of cell cycle regulation of telomerase activity in human cells. *Proc Natl Acad Sci USA* **94**: 10687–10692

Holt SE, Glinsky VV, Ivanova AB and Glinsky GV (1999) Resistance to apoptosis in human cells conferred by telomerase function and telomere stability. *Mol Carcinog* **25**: 241–248

Huschtscha LI, Bartier WA, Ross CE and Tattersall MH (1996) Characteristics of cancer cell death after exposure to cytotoxic drugs in vitro. *Br J Cancer* **73**: 54–60

Iguchi H, Morita R, Yasuda D, Takayanagi R, Ikeda Y, Takada Y, Shimazoe Y, Nawata H and Kono A (1994) Alterations of the *p53* tumor suppressor gene and Ki-*ras* oncogene in human pancreatic cancer-derived cell lines with different metastatic potential. *Oncol Rep* **1**: 1223–1227

Kerr JF, Winterford CM and Harmon BV (1994) Apoptosis. Its significance in cancer and cancer therapy. *Cancer* **73**: 2013–2026

Kim NW, Piatyszek MA, Prowse KR, Harley CB, West MD, Ho PL, Coviello GM, Wright WE, Weinrich SL and Shay JW (1994) Specific association of human telomerase activity with immortal cells and cancer. *Science* **266**: 2011–2015

Kondo Y, Kondo S, Tanaka Y, Haqqi T, Barna BP and Cowell JK (1998) Inhibition of telomerase increases the susceptibility of human malignant glioblastoma cells to cisplatin-induced apoptosis. *Oncogene* **16**: 2243–2248

Ku WC, Cheng AJ and Wang TC (1997) Inhibition of telomerase activity by PKC inhibitors in human nasopharyngeal cancer cells in culture. *Biochem Biophys Res Commun* **241**: 730–736

Lee HW, Blasco MA, Gottlieb GJ, Horner JW, Greider CW and DePinho RA (1998) Essential role of mouse telomerase in highly proliferative organs. *Nature* **392**: 569–574

Leteurtre F, Li X, Gluckman E and Carosella ED (1997) Telomerase activity during the cell cycle and in gamma-irradiated hematopoietic cells. *Leukemia* **11**: 1681–1689

Lionetto R, Pugliese V, Bruzzi P and Rosso R (1995) No standard treatment is available for advanced pancreatic cancer. *Eur J Cancer* **31A**: 882–887

Lock RB and Stribinskiene L (1996) Dual modes of death induced by etoposide in human epithelial tumor cells allow Bcl-2 to inhibit apoptosis without affecting clonogenic survival. *Cancer Res* **56**: 4006–4012

Lowe SW, Bodis S, McClatchey A, Remington L, Ruley HE, Fisher DE, Housman DE and Jacks T (1994) *p53* status and the efficacy of cancer therapy in vivo. *Science* **266**: 807–810

Lowe SW, Ruley HE, Jacks T and Housman DE (1993) p53-dependent apoptosis modulates the cytotoxicity of anticancer agents. *Cell* **74**: 957–967

Meyerson M, Counter CM, Eaton EN, Ellisen LW, Steiner P, Caddle SD, Ziaugra L, Beijersbergen RL, Davidoff MJ, Liu Q, Bacchetti S, Haber DA and Weinberg RA (1997) *hEST2*, the putative human telomerase catalytic subunit gene, is up-regulated in tumor cells and during immortalization. *Cell* **90**: 785–795

Mizumoto K, Rothman RJ and Farber JL (1994) Programmed cell death (apoptosis) of mouse fibroblasts is induced by the topoisomerase II inhibitor etoposide. *Mol Pharmacol* **46**: 890–895

Morin GB (1989) The human telomere terminal transferase enzyme is a ribonucleoprotein that synthesizes TTAGGG repeats. *Cell* **59**: 521–529

Nakamura TM, Morin GB, Chapman KB, Weinrich SL, Andrews WH, Lingner J, Harley CB and Cech TR (1997) Telomerase catalytic subunit homologs from fission yeast and human. *Science* **277**: 955–959

Nakayama J, Saito M, Nakamura H, Matsuura A and Ishikawa F (1997) *TLP1*: a gene encoding a protein component of mammalian telomerase is a novel member of WD repeats family. *Cell* **88**: 875–884

Niederhuber JE, Brennan MF and Menck HR (1995) The National Cancer Data Base report on pancreatic cancer. *Cancer* **76**: 1671–1677

O'Connor PM, Jackman J, Bae I, Myers TG, Fan S, Mutoh M, Scudiero DA, Monks A, Sausville EA, Weinstein JN, Friend S, Fornace AJ Jr and Kohn KW (1997) Characterization of the *p53* tumor suppressor pathway in cell lines of the National Cancer Institute anticancer drug screen and correlations with the growth-inhibitory potency of 123 anticancer agents. *Cancer Res* **57**: 4285–4300

Okamoto-Kubo S, Nishio K, Heike Y, Yoshida M, Ohmori T and Saijo N (1994) Apoptosis induced by etoposide in small-cell lung cancer cell lines. *Cancer Chemother Pharmacol* **33**: 385–390

Perego P, Giarola M, Righetti SC, Supino R, Caserini C, Delia D, Pierotti MA, Miyashita T, Reed JC and Zunino F (1996) Association between cisplatin resistance and mutation of *p53* gene and reduced bax expression in ovarian carcinoma cell systems. *Cancer Res* **56**: 556–562

Reed JC, Miyashita T, Takayama S, Wang HG, Sato T, Krajewski S, Aime-Sempe C, Bodrug S, Kitada S and Hanada M (1996) BCL-2 family proteins: regulators of cell death involved in the pathogenesis of cancer and resistance to therapy. *J Cell Biochem* **60**: 23–32

Sato N, Mizumoto K, Kusumoto M, Niiyama H, Maehara N, Ogawa T and Tanaka M (1998) 9-Hydroxyellipticine inhibits telomerase activity in human pancreatic cancer cells. *FEBS Lett* **441**: 318–321

Schnall SF and Macdonald JS (1996) Chemotherapy of adenocarcinoma of the pancreas. *Semin Surg Oncol* **23**: 220–228

Shay JW and Bacchetti S (1997) A survey of telomerase activity in human cancer. *Eur J Cancer* **33**: 787–791

Suehara N, Mizumoto K, Muta T, Tominaga Y, Shimura H, Kitajima S, Hamasaki N, Tsuneyoshi M and Tanaka M (1997*a*) Telomerase elevation in pancreatic ductal carcinoma compared to nonmalignant pathological states. *Clin Cancer Res* **3**: 993–998

Suehara N, Mizumoto K, Tanaka M, Niiyama H, Yokohata K, Tominaga Y, Shimura H, Muta T and Hamasaki N (1997*b*) Telomerase activity in pancreatic juice differentiates ductal carcinoma from adenoma and pancreatitis. *Clin Cancer Res* **3**: 2479–2483

© 2000 Cancer Research Campaign

Thompson CB (1995) Apoptosis in the pathogenesis and treatment of disease. *Science* **267**: 1456–1462

Vaziri H and Benchimol S (1998) Reconstitution of telomerase activity in normal human cells leads to elongation of telomeres and extended replicative life span. *Curr Biol* **8**: 279–282

Warshaw AL and Fernandez-del Castillo C (1992) Pancreatic carcinoma. *New Engl J Med* **326**: 455–465

Zhu X, Kumar R, Mandal M, Sharma N, Sharma HW, Dhingra U, Sokoloski JA, Hsiao R and Narayanan R (1996) Cell cycle-dependent modulation of telomerase activity in tumor cells. *Proc Natl Acad Sci USA* **93**: 6091–6095

*© 2000 Cancer Research Campaign*
